# On the Anatomical Characters of Some Adventitious Structures

**Published:** 1830-07-01

**Authors:** 


### On the Anatomical Characters of some Adventitious Structures. By *Dr. Hodgkin.*

[Medico-Chirurgical Transactions, Vol. XV. Part II.]

Our readers are aware of our sentiments respecting the value of morbid anatomy, and we need not repeat them at any length now. None can appreciate more highly than we do the real advantages which have been and will be derived from this source, one of the most pregnant with *certain* information of any within the range of the various departments of medical learning. But, as generally happens, there are some of an enthusiastic temper and a theoretical turn, who look upon morbid anatomy as the elixir vitæ, the arcanum of our science, which is to solve all mysteries, dispel all doubts, raise the modern physician with his scalpel and dissecting-case immeasurably beyond the disciple of Hippocrates in former centuries, and in short can transform the most uncertain of all species of human knowledge into the sober and positive reality of a mathematical proposition. We appeal to the several journals of the day for evidence of the accuracy of the character we have given of this wild and visionary party, a character which might readily have been looked on as caricature. It is not so, however; and on more than one occasion we have raised our voices against individual disciples of this modern and credulous sect. We would then encourage the prosecution of morbid anatomy by every means in our power, but let those who do so bear in mind this caution, that the appearances visible to the eye and palpable to the touch are not *always* the disease he has been treating ineffectually during life, but its sequelæ; they are not the emblems of what it is his business, and should be his aim, to cure, but the gloomy trophy of a victorious malady, the signs that he has failed in arresting the morbid actions by which their organic lesions were produced.

Bearing these facts in mind, the cultivator of morbid anatomy will improve himself and his profession, and the *facts* which he accumulates, if carefully observed and faithfully recorded, will give to his writings a perpetuity of fame and value, which the views of systematists and speculations of theorists cannot possibly maintain. The works of Morgagni will be referred to so long as medicine and science shall endure. Dr. Hodgkin, whom we are happy to call our friend, deservedly holds a distinguished place as a morbid anatomist, for great opportunities, seconded by equal or greater diligence, have given his opinions in this department the weight and measure of authority. The "Memoir on some Adventitious Structures," in the last volume of the Medico-chirurgical Transactions, is one that we would earnestly recommend to the attention of our readers. Our limited space and the practical nature of this Journal must, we fear, prevent our entering on the analysis of the paper, and to attempt a review of it would be much such a farce, as professing to review a work on trigonometry in half a dozen columns. We confess that we are deficient in the cool assurance and consummate gravity, which enable critics to knock off a review in a page of large print, and *compress* the pith of a dense octavo in a short remark and a long quotation. In the present case, then, we can neither promise a review nor an analysis, but shall merely offer one or two samples of the Doctor's fare, to serve, like the quails to the Scotchman, for a whet to his reader's appetite. Unlike the Scotchman, however, we cannot doubt that the said reader will find his appetite not a whit the worse for the dejeuné.

Dr. Hodgkin divides the *adventitious* serous membranes or serous cysts into two classes. The first comprises those which are simple, for the most part solitary, and not possessed of the remarkable property of giving origin to similar new growths. Such are the cysts in the plexus choroides, female mamma, and occasionally in the ovaries, &c. The second class consists of those which are capable of producing other cysts of a similar character with themselves, or morbid growths, which, if they do not present, strictly speaking, the character of cysts, are nevertheless referrible to the same type or mode of formation. Such are most frequent in the ovaria. At the time when a post-mortem examination is usually obtained the cure has been long in progress, and the cyst has to a great degree lost the character of a serous membrane. It now presents the following appearances:

"Its parietes appear to be rather fleshy, or coriaceous, than membranous; and the internal surface becomes more or less generally roughened, as if by ulceration or abrasion. The most important feature which it presents, is the appearance of tumours and elevations dispersed more or less thickly over the internal surface, and which, notwithstanding the very great variety which at the first view they seem to present, are nevertheless referrible to one general mode of formation.

"I shall commence with the description of that form which is intermediate between the two extremes; not merely because I shall more readily proceed from this as a standard to the explanation of its modifications, but also because the peculiar structure is, in this form, the most distinct and intelligible.

"In this form we observe on the interior surface of the principal cyst elevations more or less rounded, and of various sizes, projecting into the interior of the cavity, and covered by a membrane, which is continuous with the lining of the principal sac.

"On making an incision into these tumours, we find that they also consist of cysts of a secondary order, filled by a secretion, often serous, but almost as frequently mucous. It is not, however, merely by this secretion that these cysts are filled. On looking more minutely into them, we shall generally find, that from one or more points on the interior of these cysts there grows a cluster of other or tertiary cysts, upon which is reflected the lining membrane of the cyst in which they are contained. Cysts of the secondary order not unfrequently afford as complete specimens of a reflected serous membrane as either the pericardium or the tunica vaginalis, the lining membrane of the containing cyst corresponding to the reflected portion, as that covering the contained bunch of cysts does to the close portion.

"The proportion which the contained cysts bear to the cavity of the membrane reflected over them, is extremely various. Sometimes the fluid, especially when it is of a serous character, nearly fills the containing cyst, whilst the bunch of cysts is of very inconsiderable size. At other times, the superior cyst is almost entirely filled by those of the inferior order; in which case we may generally find, that the nodulous or tuberose elevations, which we may have observed on the exterior of the containing cyst, are occasioned by the unequal development of the contained cysts; for those which have grown most rapidly and have attained the largest size, forcibly dilating that part of the cyst which is reflected over them, produce a kind of hernia at that part. It sometimes happens, that the distension occasioned by the growth of the contained cysts is sufficient not only to disturb the even surface of the containing cyst, but actually to produce a rupture, which admits both of the escape of its fluid contents, and of the unrepressed growth of the secondary or tertiary cysts, which took their origin from its internal surface. The inferior cysts themselves are found to contain a serous or mucous secretion, and very often to produce another order of cysts, possessing the same character with themselves. It is certainly by no means surprising that these cysts of different orders, which sometimes present the appearance of delicate and pellucid sacs filled with clear and colourless serum, and possessed of the astonishing power of giving rise to an almost innumerable multitude of cysts presenting the same character with themselves, should at the first view have been confounded with true hydatids; but it is no less surprising, that a little careful inspection did not at once irrevocably remove the delusion.

"First.—Because the bunches or clusters of secondary cysts are invariably and permanently attached to and continuous with the internal surface of the superior cysts, in which they are contained; and,

"Secondly.—Because delicate vessels are seen ramifying from the one upon the other." 279.

In another variety the cysts are characterised by slender peduncles, and in a third by broad and extended bases.

"The pedunculated cysts are either produced singly, but in the closest approximation from a particular part of the containing cyst, or they may be attached to it by a common peduncle, from which the proper peduncle of each proceeds. These elongated productions sometimes become highly vascular, and, in the defect of an internal secretion, contribute largely to that which occupies the sac into which they project. Sometimes, on the contrary, they are very feebly organized, and appear ultimately to lose their vitality, in consequence of the kind of strangulation which they receive at the narrow neck by which they are attached to the containing cyst.

"It would appear, that the pedunculated cysts and filaments which have thus lost their vitality, are a pretty frequent source of irritation to the serous membrane reflected over them, which constitutes the containing cyst; the product of the inflammation thus excited is of the inorganizable kind, and often forms a thick and grumous substance, which sometimes may be washed out from the bunches of filaments, but at other times these come away with it, in the form of shreds.

"The bunches of slender pedunculated cysts, and the tufts of filaments almost resembling tassals, sometimes proceed at once from the inner surface of the principal cyst, but they are more frequently met with growing from the interior of the secondary cysts." 283.

The secondary cysts having a broad attachment and flattened form are collected in clusters on the parietes of the superior cyst, constitute shut cavities, acquire at times a considerable size, contain in some instances a serous and in others a mucous secretion, and produce in their parietes inferior orders of cysts, having like themselves broad bases and flattened forms.

"From the extent of their bases, the secondary cysts in this variety occupy proportionably a much larger space on the internal surface of the containing cyst, and by their development, although they increase its size, they seem more completely to encroach on its particular cavity. In cutting into a tumour composed of this form of cysts, we may find, it is true, several cavities of considerable size, but we shall probably not find the greater part of the fluid collected into one particular cavity. Hence, in this variety of ovarian dropsy, fluctuation is often obscure, and the relief afforded by paracentesis only partial and trifling. I am not aware that the secondary cysts, in this variety, ever lose their vitality from defect of nutrition, but if such an effect be ever produced, it cannot be the result of so limited and partial a cause as that which we have seen to operate in the preceding variety. There is another point of difference no less worthy of remark as distinguishing this variety from the two preceding, that is to say, from the standard and the pedunculated variety, which consists in the arrangement of the subordinate parts. In the two last-mentioned forms, in consequence of the limited extent of the spots, whence the secondary productions take their rise, they necessarily acquire somewhat of a radiated arrangement, whereas, in the variety with which we are at present occupied, it is difficult, if not impossible, to reduce its internal structure to any definite arrangement." 285.

Although we may observe these three well-marked forms in ovarian serous cysts of the second class, and though for the most part each individual case more particularly affects one or other of these forms, it occasionally happens that two or all may be found in the same superior cyst, whilst even then one form seems to predominate. With some remarks on the etiology of these ovarian tumours we must conclude.

"There seems to be an hereditary disposition in some females to the production of the serous ovarian cysts. Even in these cases they are mostly unaccompanied with any constitutional taint, that is to say, other parts of the body are not simultaneously affected with similar productions. It is even more common for one ovary to be singly affected, than for both to give origin to this form of cysts; nevertheless it does sometimes happen that we meet with cases of double ovarian dropsy, but in many, if not in most of these, there is likewise a complication with some one of the diseases commonly called Malignant, of which I am about to speak. It is by no means easy to say what are the exciting causes of this form of ovarian dropsy. Though in many instances the patients refer the commencement of the disease to parturition; yet it is far from being uncommon for unmarried or barren women to labour under this affection. The tumours and growths allied to ovarian dropsy, of which I shall presently speak, as formed in other parts of the body, can often be referred to some mechanical injury, but in the case of organs, which appear to be so well protected as the ovaries, it is more difficult to conceive the possibility of such an exciting cause.

"Something may possibly be ascribed to the natural and periodical changes which these organs in common with other parts of the female genital system doubtless undergo." 286.

The following remarks on the mode of examining malignant tumours by means of sections are deserving attention.

"The tumours of the description of which I am now speaking have a more or less rounded form. On making the section of them they present various appearances, but are all more or less divided by septa, which affect sometimes a radiated form, and at others a cellular character. Both of these characters have been insisted on by many writers on this subject; but I believe the differences which have been observed in many instances depended on the direction in which the sections were made.

"The mode of examination by means of sections, if it be the only one employed, is not better adapted for the investigation of these tumours than for that of the brain. The objection to it is increased by the plan of immersing the specimen in alcohol, which is sometimes had recourse to for the purpose of hardening the parts. By this measure the fluids are coagulated, and the transparent parts rendered opaque: we consequently destroy two of the most important characters which assist the examination, by marking the boundaries of structure and arrangement. It is on this account that almost all the preparations which I have made myself, or seen made by others, are more or less unsatisfactory, and, even in the most successful cases, fall incomparably short of the inspection of the recent specimen. If we carefully dissect down to the surface of one of these tumours, we shall usually find that it has a capsule or covering, which has, I believe, generally been supposed to consist of the altered and condensed cellular membrane of the parts which have given way before the growth of the tumour. This idea is probably correct with respect to the unequally thick external part of the capsule; but if we dissect carefully, and examine those tumours in which the process of decay has either not commenced, or has made very little progress, we shall find that surface which is next to the mass of the tumour more or less smooth and even, and on raising it we find that it is reflected over one or more somewhat pyriform bodies, attached by a base, which is generally narrow and peduncular to some part of the circumference of the inclosing capsule. Unless the tumour is very small, it is much more common to find several rather than a single body of this kind, and as there is often little, if any, fluid intervening between them and the enclosing capsule, their form is somewhat modified by their mutual pressure. Sometimes, though more or less closely applied to each other, these pedunculated bodies are perfectly detached at their sides, and may, consequently, be readily traced to the point which forms the common origin of their peduncles. At other times, these bodies are so adherent amongst themselves, and the membrane covering them is so tender and delicate, that without very great care the arrangement of their structure may be overlooked, in consequence of the pedunculated bodies being broken or torn through in a different direction from that to which their mode of formation would naturally dispose them.

"It must be sufficiently obvious that the appearance presented by the section of a tumour, such as I have just described, must be very materially affected by the direction in which the section is made. If it pass through or near to the point at which the pyriform bodies are attached to the enclosing cyst, it must nearly correspond with the direction which some of these bodies take towards the circumference, and their edges will consequently be seen in the form of radiating lines. On the other hand, if the section be made more or less nearly transversely to the axes of these bodies, their sections will convey the idea of cells of various shapes. If we continue dissecting and raising the outer cyst, forming the reflected membrane which covers the radiating pedunculated bodies, we shall generally find, that on one or more sides it dips down deeply into the mass of the tumour, and forms a part of the septum which separates the one packet of pedunculated bodies from the others which generally concur to form the mass of the tumour, for it comparatively rarely happens that the tumour is composed of a single cyst filled with pedunculated bodies. On examining the different encysted packets of pedunculated bodies which compose the tumour, we shall often find some indication of their having taken their origin from nearly the same spot, which is generally the most indurated part of the tumour. We may likewise observe, that the different secondary tumours or encysted bundles of pedunculated bodies are in very different stages of progress." 297.

We said that this could only be a notice of Dr. Hodgkin's Memoir and we have kept our word. We wish the worthy Doctor health to pursue his perilous avocations, and opportunities of laying the profession under obligations by communicating the results of his labours to his brethren.

